# Updates on Treatment Modalities for Primary Rhegmatogenous Retinal Detachment Repair

**DOI:** 10.3390/diagnostics14141493

**Published:** 2024-07-11

**Authors:** Sofija Davidović, Siniša Babović, Aleksandar Miljković, Svetlana Pavin, Ana Bolesnikov-Tošić, Sava Barišić

**Affiliations:** 1 Department for Ophthalmology, Medical Faculty, University of Novi Sad, Hajduk Veljkova 1–9, 21000 Novi Sad, Serbia; aleksandar.miljkovic@mf.uns.ac.rs; 2 University Eye Clinic, University Clinical Center of Vojvodina, Hajduk Veljkova 1–9, 21000 Novi Sad, Serbia; sinibab@gmail.com (S.B.); svetlanapavin@gmail.com (S.P.); savabarisic@gmail.com (S.B.); 3 University Clinic for Anesthesiology, University Clinical Center of Vojvodina, Hajduk Veljkova 1–9, 21000 Novi Sad, Serbia; srckovs@gmail.com

**Keywords:** rhegmatogenous retinal detachment, surgical options, equatorial cerclage, scleral buckling, pneumatic retinopexy, pars plana vitrectomy

## Abstract

Rhegmatogenous retinal detachment, a severe eye condition, presents anatomic separation of the neurosensory retina from its outermost layer—the retinal pigment epithelium. Early recognition of this relatively common finding and proper referral of patients to the retinal surgery department is essential in order to minimize its consequent possible severe reduction in vision. Several major surgical methods for the repair of primary rhegmatogenous retinal detachment have been in use over the last several decades, and they all aim to find and close the break in the retina that has caused the detachment. Surgery can be performed as pneumatic retinopexy, pars plana vitrectomy, and/or episcleral surgery (buckling). General surgical trends for reattaching the retina include moving from extraocular to intraocular surgery and from bigger gauge to smaller gauge via minimal invasive vitrectomy surgery (MIVS), with implementing shorter-lasting intraocular tamponades. Surgical options for rhegmatogenous retinal detachment treatment nowadays emphasize gaining retinal reattachment, preferably with one surgery and with minimum damage to the eye. The procedure should not bring secondary eye conditions and complications with severe impairment of visual acuity, and it should be performed on as much as a smaller budget, with possibly peribulbar anesthesia, enabling the patient the quickest possible recovery. It should be adjusted to the patient’s condition, not to the surgeon’s skills or preferences.

## 1. Introduction

Retinal detachment (RD) is the anatomic detachment of the neurosensory retina from its outermost layer, retinal pigment epithelium (RPE). The full-thickness retinal break, which is a main step in the pathophysiology of rhegmatogenous retinal detachment (RRD), enables access of liquefied vitreous to the subretinal space [1,2,3].

An estimated frequency of RRD occurrence is 1 in 10,000 people per year [4,5,6]. Depending on the scale of clinical presentation and accessibility of patients to prompt ophthalmo-surgical care, there is a variable rate of patients successfully operated [7]. Some data suggest that approximately 95% of all RRDs are being treated. Despite that, RRD still presents a serious eye condition that can bring blindness or severe impairment of visual acuity [8,9,10].

In the modern world, with prolonged life spans due to improvement of life quality and better health care, with more and more frequent cataract and refractive surgery, growing reports on the incidence of myopia, and better diagnostic tools available in ophthalmology, retinal detachment has become more frequently encountered eye condition in everyday practice [11,12,13]. This serious eye condition is encountered by general ophthalmologists, general practitioners, and other specialties, and its early recognition and prompt referral to posterior segment ophthalmologists and the retinal surgery department are essential [14].

### 1.1. History of Surgical RRD Treatment

Early attempts at achieving retinal detachment repair before the 20th century were hardly successful. Renewed ophthalmologist Gonin in 1918 brought new insights into retinal detachment pathophysiology, which previously had not been fully understood. The role of the retinal break-hole and the significance of its ophthalmoscopic finding and closing were presented in his work. He introduced methods of transscleral cauterisation, and a procedure named ignipuncture [15,16]. Yet Custodis, in 1949, invented certain episcleral buckling procedures [17]. Nowadays, alike, buckling surgery, with a report of its relatively high anatomic repair, as high as 80–90% (for the time mentioned), was implemented by Schepens [18,19] and his colleagues. Episcleral, i.e., scleral buckling surgery, has remained the gold standard for RD repair for many years [20].

In the early 1970s, Machemer brought to surgical life the idea and method of vitrectomy. In the very beginning, vitrectomy as the surgical option for RRD repair was used for more complicated or recurrent RRD. The first vitrectomy method for primary simple and short-standing retinal detachment occurred in 1985. Over the last decade of the 20th century, the vitrectomy technique has become more frequently used for primary uncomplicated RRD repair, as a technique alone or in combination with scleral buckling. Pneumatic retinopexy, another method for the treatment of uncomplicated RRD, was introduced in 1986 by the work of Hilton and Grizzard. The frequency of clinical use of pneumatic retinopexy varies in different regions. Due to its features, like being relatively easy and quick (even office-based) to perform, it is the preferred method of certain retinal surgeons, especially in the United States. However, in Europe and other parts of the world, it is less popular [21].

### 1.2. The Aim of the Paper

Comparing the anatomic and functional outcomes of surgical procedures used for retinal detachment repair and treatment of other retinal pathologies is still one of the main topics in surgical meetings. It is also important in clinical practice today since scleral buckling and pars plana vitrectomy have advantages and disadvantages. The importance of surgical skills and case-dependent decisions for a combination of two procedures at the same time may also be an option [22,23].

Ophthalmic surgery is high-end surgery, and it keeps implementing new and modern surgical techniques in everyday work, including constantly evolved and small gauge surgical instruments, enhanced operative access to retinal structures, and improved visualization. Newer and faster cutting vitrectomy machines with preferable use of short-term intraoperative tamponades brought safer surgical work and better anatomic and functional prognosis of RRD surgery, also improving life quality and decreasing lifestyle limitations of our patients. Still waiting for uniform agreement among retina specialists on the first surgical option for retinal reattachment has yet to be defined [24,25,26,27,28].

The aim of this paper was to make an overview of *the state-of-the-art* approach to RRD treatment, referring to its origins and first surgical ideas that have eventually brought our clinical practice to where it is now. The review paper that is presented here is based on data from the literature available. Apart from the theoretical introduction, we aimed to analyze certain parts of available scientific evidence on outcomes of PR, PPV and SB for management of RRD from the literature. We searched MEDLINE and PUBMED to identify studies that compared PR, PPV and SB in primary RRD treatment. Comparative studies, randomized controlled trials and observational studies investigating PR, PPV and SB for RRD repair from approximately the last 20 years were analyzed, and the results of several of them were included in the paper. The searched endpoints were final best-corrected visual acuity, retinal reattachment rates, single surgery success, and type of most frequent complications and adverse events of each of the mentioned procedures. Studies including other topics than primary RRD, and other analyzed criteria were not presented here. The choice of the data on RRD treatment available is vast, leading to the conclusion that further studies, including our consecutive work and other new available data presentations, should follow.

The figures that are included in the paper were made during routine primary RRD surgery (one surgeon), performed at the University Eye Clinic, Clinical Center Vojvodina, Novi Sad, Serbia*. Screen grabs were taken from a movie recorded with the integrated camera of a Zeiss OPMI Lumera operating microscope (Carl Zeiss Meditec AG, Jena, Germany) and subsequently edited and collaged using the Preview app within macOS (Apple Inc., Cupertino, CA, USA, macOS Sonoma 14.5).

## 2. Relevant Sections

### 2.1. Pathophysiology and Mechanisms of RRD

Retinal detachment can be divided into four major categories. It can be rhegmatogenous, tractional, combined tractional and rhegmatogenous, and exudative [29], with RRD being by far the most frequent form of it. As already mentioned, RRD develops from a full-thickness retinal break and is the main topic of this report.

The physiological aging process in the eye includes biochemical, molecular, and histological changes in vitreous structure, its consequent liquefaction, syneresis, and formation of big *pockets* of the vitreous gel, which are called lacunae [30]. This is how physiological, so-called acute posterior vitreous separation (posterior vitreous detachment) occurs. In certain predisposed eyes, it can lead to further pathological conditions due to retinal tear formation and consequent RRD [31].

Specific anatomic points of firmer adherence of vitreous gel to the retina are known. Such as are optic nerve head, retinal blood vessels, peripheral retina, and vitreous base insertion, and sometimes even the macular region. As the posterior vitreous separates from the inner retinal surface, it can lead to retinal tear formation, especially in those regions with emphasized vitreal adherence. Excessive and abnormal vitreal traction on retinal tissues is most frequently happening at posterior insertions of the vitreous base and in the areas of lattice degeneration [32]. Typical flap-shaped horseshoe tears occur as a dangerous pathophysiological outcome. 

With time, further replacement of liquified vitreous gel in the subretinal space through a retinal tissue defect (tear or hole) leads to neurosensory retina disinsertion (detachment) from the underlying retinal pigment epithelium [33].

The age of the patient, his refractive condition (myopia), previous eye surgeries, presence of longer-standing blood in the vitreous gel, and traumatic origin of RRD may sometimes influence the preferred method of first surgical approach to RRD treatment due to the amount of posterior vitreous gel separation. For the clinical presentation of retinal detachment with almost complete posterior vitreous separation, especially in pseudophakic eyes, vitrectomy is the most favorable choice for repair. For some instances of retinal detachment with no complete posterior vitreous detachment and in younger eyes with visible peripheral retinal tear, scleral buckling may be the primary type of surgical RRD treatment.

### 2.2. Methods of Surgical Procedures for RRD

Rhegmatogenous retinal detachment, as a clinical feature, can present with different options regarding the stage of RRD, retinal tear number and location, scar tissue formation (proliferative vitreoretinopathy (PVR)), number of retinal tissue quadrants affected, macula presentation (macula on or macula off in RRD), vitreous features, etc. Therefore, it presents heterogeneous retinal disease, which may vary in many clinical aspects, which is the main factor influencing our surgical approach and decision.

Depending on its clinical aspect, RRD is an eye condition that may be treated with several surgical approaches, including pneumatic retinopexy (PR), episcleral surgery (scleral buckling procedure and/or encircling band implementation (SB)), pars plana vitrectomy (PPV), or pars plana vitrectomy with episcleral surgery. Term scleral buckling in practical surgical terms has several considerations. It may consist of suturing of encircling band element, radial episcleral element, or attaching both on the surface of the sclera. It may include drainage of subretinal fluid (SRF) or not, and it may consist of intravitreal gas injection, or it can be performed as a scleral buckling procedure without gas insufflation. 

Despite continued advances in vitreoretinal surgery and numerous data analyzed in the literature, 1st line choice of surgery for primary (RRD) repair still acts as an important point of discussion for vitreoretinal surgeons worldwide [34]. Nowadays, the most commonly performed surgical procedure in the repair of RRD is pars plana vitrectomy, while scleral buckling is less frequently used [35]. Both types of surgeries have the primary goal, and that is to succeed in reattaching the retina to the retinal pigment epithelium surface, relieving vitreoretinal traction that has caused a disease [36].

### 2.3. Scleral Buckling

**Figure 1:**
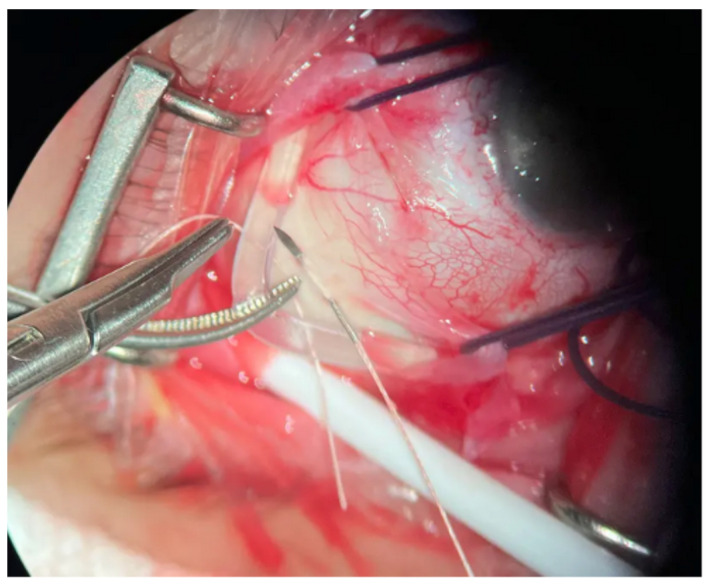
Scleral buckling procedure in primary RRD treatment with an episcleral encircling band suturing *.

Scleral buckling (SB) historically has emerged as the first surgical procedure for RRD repair, therefore having the longest available published follow-up data [37]. Scleral buckling surgery consists of placing and suturing an episcleral implant (sponge) with the potential use of single or multiple sponges, and/or encircling element placement (silicon band), with or without external subretinal fluid drainage, and with or without intraocular gas injections (Figure 1).

In some instances, SB is the dominant choice of primary retinal detachment repair. Indications for SB are, in the 1st place, related to age (young) and phakic status of the patient, number (preferably one), size and location of the retinal break(s), and escorting technical issues connected with placement of the buckling elements themselves (Table 1) [34].

Contraindications for SB are most often multiple retinal tears (that cannot be covered with episcleral implants and/or encircling band), very posterior retinal breaks, proliferative vitreoretinopathy grade C, significant vitreous opacities, thin sclera, prior strabismus or glaucoma filtration/implantation surgery, and other eye conditions [7].

### 2.4. Pneumatic Retinopexy

Pneumatic retinopexy (PR) is one of the RRD surgery options that can be performed in an ambulatory–office setting. In terms of costs, it is the least expensive surgery and the only retinal reattachment surgery that does not necessarily require the patient’s preoperative preparation and surgical theater.

According to the available data, the single-operation success rate (SOSR) for PR, which is derived from published case series of RRD patients, is about 70% [38,39]. Thus, these data suggest that PR’s relatively low retinal reattachment rate after only one surgical procedure is actually its most significant disadvantage compared to other RRD techniques. 

Proper preoperative patient selection is the most important prognostic factor for PR. A better prognosis for this type of retinal RRD surgery is predicted for patients with phakic RDs, fresh, superior retinal breaks, and one or several closely situated breaks [40,41]. 

Patients with RD caused by retinal tears localized between 11 and 1 o’clock and along the horizontal meridians appear to be the best candidates for primary surgical retinal reattachment repair. According to the literature, breaks in the oblique meridians may be more challenging to close, leading to the necessity of repeated surgical maneuvers [21,25]. 

Once more to emphasize, excellent patient selection remains of most significant importance in gaining successful anatomic and visual outcomes in RRD treated with pneumatic retinopexy. One of the main disadvantages of this type of retinal surgery is retinal redetachment, often leading to quicker PVR formation, sometimes macular pucker formation (though less frequently than in PPV with SO tamponade), and much worse rhegmatogenous retinal redetachment surgery prognosis. The following SB or PPV procedure should be prompt, and new retinal surgery in these cases should be performed in consequent three to five days after the primary pneumatic retinopexy surgery in order to minimize excessive postoperative inflammation and proliferative vitreoretinopathy resulting in the first place from cryotherapy. Other complications of PR are less common [42,43,44,45,46]. 

### 2.5. Pars Plana Vitrectomy 

**Figure 2:**
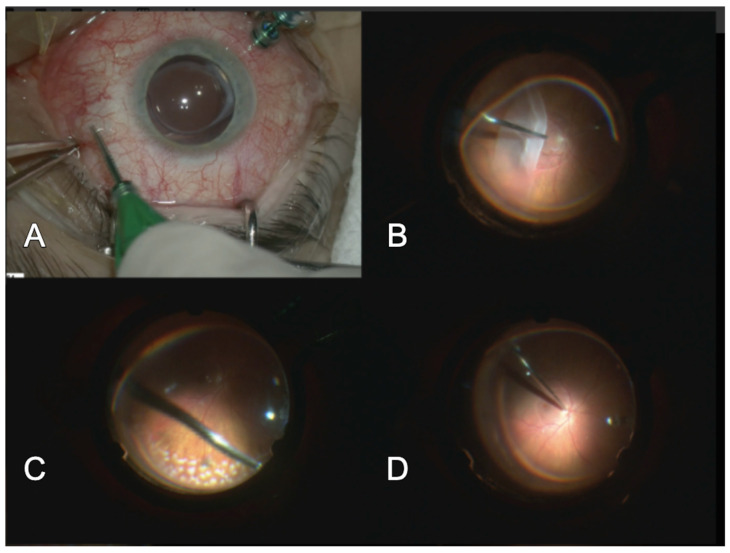
Pars plana vitrectomy procedure in primary RRD treatment with the use of perfluorocarbon and silicone oil tamponade. (**A**) 3 port PPV procedure. (**B**) RRD with the instillation of perfluorocarbon to flatten the retina. (**C**) endolaser fotocoagulation of the retina with temporary perfluorocarbon tamponade. (**D**) reattached retina with silicon oil tamponade *.

Modern ophthalmic surgery puts pars plana vitrectomy (PPV) without any doubt in the first place as a surgical choice for primary RRD repair. It has immense popularity worldwide, especially for RD in pseudophakic cases. Technical features of PPV have brought it to its popularity, because it brings more comfort and postoperative benefit both for the patient as well as for the surgeon. PPV surgery is related to the latest technical advances in instrumentation; it is performed with smaller-gauge transconjunctival and sutureless wounds (so-called minimal invasive surgical procedures—MIVS). PPV machines are continuously implementing higher and higher vitrectomy cutting rates, enabling safer and faster retinal surgeon’s work, therefore reducing the risk of iatrogenic retinal tear and increasing the number of surgeries in a day. Very innovative and important are wide-angle and 3D heads-up viewing systems, which are being more and more used worldwide for RRD (and other retinal or cataract) surgery, sometimes containing intraoperative optic coherence tomography (OCT) as additional useful surgical tools for retinal layers observation [47] (Figure 2).

PPV, by its intraocular approach and, in most cases, good visualization of the retina, presents surgical options with predominant advantages compared to SB. Most important is the location of previously known or detection of new retinal tears, especially enabled with peripheral scleral indentation during surgery. In that way, preoperatively missed retinal breaks and retinal tears can be detected on the operating table. Sometimes, opacities of lens capsules, intraocular lens optic or haptic margins, corneal opacities, or insufficient intraoperative mydriasis can make the detection of the very peripheral breaks complicated. The most common types of intraocular tamponading agents that keep the attached retina in place are expandible gases (SF6 and C3F8) and silicon oil (SO). Rarely heavy-density (HD) silicon oil can be used for inferior breaks. 

Another advantage of PPV as a method is its direct and immediate vitreous traction release from the retina, which is the primary pathogenic substrate for RRD occurrence. The removal of vitreous opacities, synechiae, intravitreal blood (promotor of PVR), and more controlled intraocular drainage of subretinal fluid is also obtained with PPV. Surgeons can use preexisting retinal defects for intraocular drainage or can create another iatrogenic break at the preferable location in the eye for safer and more convenient subretinal fluid removal. 

Furthermore, PPV is much less likely to cause significant surgically induced myopia, astigmatism, or motility disturbances, leading to motility problems and diplopia, and it is considered to be less painful than SB. Patients’ intraoperative comfort is significantly higher in PPV surgery, which is most regularly performed in peribulbar anesthesia, while the type of surgical procedure in SB surgery predominantly requires general anesthesia due to the ocular pain and muscle traction. 

On the other hand, PPV is more frequently associated with cataract formation in phakic eyes [48,49]. It may cause intraocular pressure elevation [50] (both intraoperatively with the use of heavy liquids (like perfluorocarbon) or postoperatively with expanding intraocular gas tamponades or silicon oil tamponades) [51]. There is also the possibility of new and iatrogenic retinal break formation. Intraocular gas tamponade may sometimes create excessive traction on the retinal tissue and cause anterior displacement of the lens or intraocular lens–iris diaphragm. In aphakic eyes, gas can migrate to the anterior chamber, causing the attack of secondary glaucoma [52].

Heavy liquid droplets may be retained in the eye. At the same time, after silicon oil tamponade, even after silicon oil removal in successful retinal redetachment surgery, particles of silicon oil may be discovered lifelong in all tissues of the eye and under the conjunctiva. Not-so-frequent complications of PPV include retinal incarceration into a sclerotomy, displacement of a LASIK flap after flap-based corneal surgery, retinal trauma during vitreous shaving or fluid–air exchange, displacement of the retina, and unfavorable surgical outcomes (Table 1) [53,54,55].

## 3. Discussion

Over the past few decades of RRD techniques analysis, data have shown that the benefits of PPV surgery enhanced its possible complications in patients with primary RRD, and numerous studies from different surgical centers worldwide reported good anatomic and visual results of this type of intraocular surgery [56,57]. PPV has proved to be successful also in cases with inferior breaks or with no visible breaks. Silicon oil [58] and air or gas tamponade [59,60] have broadened the surgical success of PPV surgery for primary RRD patients, though with specific postoperative positioning requirements [61]. 

Randomized clinical trials (RCTs) have contributed to a better understanding of the advantages and disadvantages of each of these three major procedures for RRD surgical repair.

The Retinal Detachment Study Group [62,63] analyzed data from 198 patients from seven surgical centers who had RD with superiorly located retinal pathology and who were randomized to the SB or PR group. They have been observed for at least six months., and the status of retinal reattachment was compared. SB-treated patients had higher SOSR (82% vs. 73%), whereas, in PR-treated patients, significantly better visual outcomes were recorded. Multiple single surgical center studies on RRD have been conducted in all parts of the world, with the subject of the comparison of SB to PR surgical approach. Most of the studies have found no statistically significant differences in either SOSR or visual postoperative success between these two (SB or PR) surgical approaches [27,64].

The Scleral Buckling versus Primary Vitrectomy in Rhegmatogenous Retinal Detachment study is one of the most important European multicenter RCTs, which was based on a comparison of two standard surgical procedures—PPV and SB. The study group introduced the term “medium-severe” RD clinical form, for which there appeared to be no apparent practical advantage in treatment between SB and PPV. In this study, on the basis of detailed fundus drawings, 28.2% of patients with RDs were qualified as patients with “medium-severe” RD. These cases were described with a clinical appearance of an average of 2.6 retinal breaks, 5.8 clock hours of retinal detachment, no visible break in 15.1% of cases, macula-on status in 42.9% of patients, bullous subretinal fluid in 15.1% cases, and vitreous hemorrhage or opacity in 7.7% of patients [65].

Forty-five surgeons in 25 centers in several European countries enrolled 416 phakic and 265 pseudophakic patients with the clinical status of “medium-severe” RD. Patients were randomized to receive SB or PPV surgical treatment. However, in some patients, PPV also included SB surgery and episcleral implant, based on the decision and preferential surgical approach to certain RRD cases. The primary study endpoint was a 1year postoperative change in best-corrected visual acuity. The secondary endpoints of the study consisted of various anatomic factors analysis, which also included the SOSR parameter. In the group of phakic patients, the mean BCVA change was significantly more significant in the SB group (SB, −0.71 logarithm of the minimum angle of resolution [logMAR], standard deviation [SD] 0.68; PPV, −0.56 logMAR, SD 0.76). In the group of pseudophakic patients, changes in BCVA showed a nonsignificant difference of 0.09 logMAR. In phakic patients, cataract progression was significantly greater in the PPV group. In the pseudophakic group, the primary anatomical success rate (defined as retinal reattachment without any secondary retina-affecting surgery) was 53.4% for SB and 72% for PPV, therefore presenting the last surgical option as statistically significantly better with the functional outcome. The average number of redetachment (secondary) retinal surgeries was 0.77 for SB and 0.43 for PPV, and in that way was significantly lower in the PPV group. Redetachment rates were 26.3% for SB and 25.1% for PPV; 52/207) in phakic patients, 39.8% for SB, and 20.4% for PPV in pseudophakic patients. 

This study group, in particular, brought to the conclusion that the SB treatment option was the preferable choice for phakic eyes with “medium-severe” RD, and PPV was the preferable surgical approach for patients with pseudophakic eyes. This critical conclusion was based on the more favorable postoperative anatomic results gained. By means of statistical modeling, the study group also reported that PPV surgery was associated with an increased risk of recurrent RD in the phakic group of patients but with a decreased risk of recurrent RD in the pseudophakic group [66].

Another study reported that SOSR in RRD surgery was assessed in 81.1% of eyes with PPV alone compared with 92.2% of eyes with combined procedure SB+PPV. Scleral buckling plus PPV showed greater SOSR than PPV alone in phakic eyes but not in eyes with a posterior chamber intraocular lens. Retinal redetachment occurred on average at 1.5 and 9 months after the initial surgery, mostly around three months after surgery [67]. 

In a comparison of outcomes of primary RRD repair of pneumatic retinopexy and scleral buckling, another study performed in a single surgical center, with its retrospective analysis of treated patients, found that the final visual outcome had no statistically significant difference between the two procedures. Single-procedure reattachment rate was significantly higher in SB (94%) than in PR (68%). The anatomical success rate was not influenced by macular involvement. The total reattachment rate, including repeated procedures, was 87% in the PR group and 94% in the SB group, without a significant difference as well. Inclusion criteria comprehend phakic patients with a single retinal break or a group of breaks in the detached retina in the same quadrant above the 8- and 4-o’clock meridians [55].

One of the studies with the same topic, optimal surgery for repair of RRD, compared outcomes of PR versus PPV for the management of primary RRD of 176 cases. Patients with RRD caused by a single retinal break or a group of breaks in detached retina within one clock hour above the 8- and 4-o’clock meridians were included. Macula-on and macula-off groups of patients were created and treated within 24 and 72 h, respectively. Visual acuity after PR exceeded that after PPV by 4.9 letters at 12 months (79.9 ± 10.4 letters vs. 75.0 ± 15.2 letters; *p* = 0.024). Mean BCVA showed to be superior for the PR group compared with the PPV group at three months (78.4 ± 12.3 letters vs. 68.5 ± 17.8 letters) and six months (79.2 ± 11.1 letters vs. 68.6 ± 17.2 letters). Vertical metamorphopsia scores were superior for the PR group compared with the PPV group at 12 months (0.14 ± 0.29 vs. 0.28 ± 0.42). Primary anatomic success at 12 months was achieved in 80.8% of patients undergoing PR versus 93.2% undergoing PPV, while secondary anatomic success was assessed in 98.7% compared to 98.6% of patients, respectively. Sixty-five percent of phakic patients in the PPV group underwent cataract surgery in the study eye within one year, compared to only 16% in the PR group, which was statistically significant. This study suggests that pneumatic retinopexy should be considered the first-line treatment for RRD in patients with fresh, superior breaks, as it brings faster and better VA recovery, induces less vertical metamorphopsia, and reduces eye morbidity when compared with PPV [43].

One more study, this time on RRD associated with giant retinal tears (at least 90° or one-quarter of the circumferential extent), systematically identified and summarized 36 clinical studies evaluating surgical techniques for the management of giant retinal tear-related RRD and risk factors affecting treatment outcomes. Four principal surgical techniques were used: pars plana vitrectomy (PPV), combined PPV and SB, SB alone, and PR. Various types of tamponades, including gas, silicone oil, and air, have been used. PPV was the most commonly used surgical technique in 33.1–100% of patients. Among the 20 studies that used PPV alone, 17 patients with preoperative PVR were included. SB alone or in combination with PPV was reported as a treatment option in 10 studies, with 2–100% of patients experiencing SB alone and 13.6–100% experiencing combined PPV and SB. Primary anatomic success was achieved with retinal reattachment via a single operation with no residual tamponade. In contrast, final anatomic success was achieved via more than one operation with no residual tamponade. Reported single-surgery anatomic success (SSAS) rates range from 65.51 to 100%. The preoperative best-corrected visual acuity (BCVA) ranged from 0.067 to 2.47 logMAR, whereas the postoperative BCVA ranged from 0.08 to 2.3 logMAR. An improvement in visual acuity was observed in 29 studies. Cataract (3.9–28.3%) was the most common postoperative complication, followed by increased IOP (0.01–51.2%) and PVR formation (0.8–31.57%). They concluded that MIVS PPV is the most common surgical technique, with silicone oil being the most frequently used tamponade in RRD repair. Risk factors for giant retinal tear-related RRD include age, gender, lens status, high myopia, PVR, initial visual acuity, the extent of the tear and RRD, and macular involvement [68]. The extent of retinal break (larger than 90 degrees up to 270 degrees, by authors) and macular involvement resulted in less favorable visual acuity outcomes after RRD surgery. Also, patients with giant retinal tears in the data tend to be younger. Young age is a significant risk factor predisposing to giant retinal tears. Tight vitreoretinal adhesions in young patients and a greater predisposition to ocular trauma could be the main causes for this finding. Trauma, high myopia, aphakia, and pseudophakia may predispose to giant retinal tears in all, especially young patients. In older subjects, these factors may lead to retinal detachment by the formation of other types of retinal breaks. Since trauma may increase the incidence of giant retinal tears, male subjects may have a higher incidence of such tears due to being more prone to trauma. The importance of giant retinal tears is that the rate of severe postoperative PVR and unsuccessful retinal reattachment is especially high despite advances in surgical technique [68].

By the study work of other authors, the safety and efficacy of PPV and PPV with additional SB in the surgical treatment of RRD were challenged. They made a meta-analysis, presenting BCVA, reattachment rates, and complications of surgery. This study included 15 661 eyes from 38 studies. The median follow-up duration was six months. The final BCVA was similar for PPV and PPV with SB. There was a significant difference in the single-operation success rate (SOSR) (88.2% versus 86.3), in this way favoring PPV with SB; however, there was no significant difference in the final reattachment rate after additional retinal surgeries. PPV required a significantly higher number of interventions in order to achieve the final anatomical result. Regarding complications, this study reported that PPV was considerably less likely to be associated with macular edema and epiretinal membrane formation. Still, the overall complication rate in the sample was similar [69].

In the literature available, available is another study that contained systematic research on the results of the efficacy and safety of PPV, SB, and PR surgical procedures in the management of uncomplicated RRDs. It presented information from three databases, with 2751 eyes included. Randomized controlled trials (RCTs) comparing RRD management options were included, and meta-analysis was performed using a random effects model. Early postoperative (up to 1 month) best corrected visual acuity was slightly better in SB 20/260 compared to counting fingers in PPV, but differences were nonsignificant at other time points. No significant difference was found for primary reattachment in comparison to those three surgical approaches. Important, PPV was connected with a lower incidence of choroidal detachment, hypotony, and strabismus/diplopia but had a higher incidence of iatrogenic breaks and cataract development/progression compared to SB. This meta-analysis concluded that PPV is associated with slower visual recovery but has similar final visual acuity and primary reattachment rate compared to SB. Combined procedures did not bring better primary reattachment rates. As well as mentioned before, heterogeneity was seen across the included trials, and further randomized trials are needed to unify the interpretation of the results [70].

Numerous publications and scientific opinions are dealing with the issues of the learning curves of PPV and SB, and comparing them. The learning curves for both surgical methods are multifactorial, and there is indeed no clear consensus on which surgical technique is easier to acquire. Some authors via the results available, though, suggest that SB may be practically more difficult retinal surgical technique, with the success being highly surgeon-dependent [68,69,70,71]. These may be one of the most important and contributing factors to the real-life situation where SB surgery has been widely replaced with pars plana vitrectomy in recent years [72].

Scleral buckling, with its particular episcleral indentation, intraoperative use of indirect ophthalmoscopy, and demanding suturing technique, has remained practically unchanged. At the same time, pars plana vitrectomy has technically immensely evolved, especially in the last several years, with the support of industry. Intraoperative use of wide-field viewing systems and heads-up surgery have made it significantly more accurate and easier, and clinical application of tools like heavy liquids (perfluorocarbon liquid, etc.) and small-incision techniques (change from 20 to 23, then gradually to even 29 gauge wound) have greatly enhanced patient safety and comfort [73,74].

It has been estimated that more than 80% of primary retinal detachment cases in the world are currently treated with vitrectomy. Some surgeons, especially in the US, report that they treat 9 of 10 cases of RRD with vitrectomy on all of their pseudophakic patients and in approximately 90% of phakic eyes. Buckling is indicated only in very few selected cases, like patients with RD with inferior temporal dialysis, esp. in phakic patients, and younger patients with post-traumatic retinal detachment [75,76]. In addition, it has been found that phakic eyes with many peripheral retinal breaks may benefit from an external, episcleral RRD approach [77].

According to the data, the real-life situation in Europe is more variable. For instance, in Germany [78,79] the proportion of scleral buckling vs. vitrectomy is estimated to be even about 40% vs. 60% [80,81], while in the United Kingdom [82,83], vitrectomy frequency has changed from 80% up to 90% of cases. Even if it is obvious that the global tendency towards doing more vitrectomy for RRD is the same [84], scleral buckling still has more percentage as the first RRD choice of treatment in Europe compared to the US [85,86,87,88].

Certain European surgeons consider that the large shift toward vitrectomy requires wider and more expert discussion and that there are clear indications for scleral buckling as the primary RRD approach that is being neglected due to PPV being the preferred and dominant surgical option. According to the data presented by several surgeons, less than 30% of RRDs nowadays in Europe are routinely scheduled for scleral buckling.

Scleral buckling has the advantage of being an extraocular procedure, and in the case of failure, surgical solutions for RRD patients can proceed toward vitrectomy (with or without an intraocular chandelier) [89]. There are also some opinions that vitrectomy should be limited to only a few selected cases such as posterior breaks, multiple or large breaks, and highly myopic eyes with a thin sclera. Philosophy, to stay away from the vitreous and spare vitreous in precisely chosen cases, is, in a way, being back in “ophthalmology fashion” by some retinal surgeons again.

Though vitrectomy for some surgeons may technically seem easier, it is an insidious part of the eye that is being operated on, and it can induce certain specific complications. In addition, data suggest that SB induces fewer cataracts, which, on the other hand, occurs in almost 80% of PPV-operated phakic eyes within one year to 2 years after surgery. SB is the preferable surgical approach in young patients with single or multiple retinal tears and an attached vitreous. Avoiding prior cataract surgery and lens sparing are priorities in these cases. SB can be the preferred surgical option in RRD in trauma cases with dialysis because a high percentage of success is achieved with a single buckle or circular episcleral implant (equatorial cerclage). SB alone can be sufficient only for certain trauma cases. Cases of severe ocular trauma often involve vitreous hemorrhage, intraocular foreign bodies, and other intraocular disturbances, making PPV or combined SB and PPV a more reasonable and more acceptable approach [77,90,91,92,93].

Last, but not least, in recent years, issues of costs of two different procedures, SB versus vitrectomy, could be put into account too, especially in moderate and low-income countries. Scleral buckling appears to be a low-budget procedure, while vitrectomy uses advanced and expensive surgical machines and particular surgical equipment. Without a doubt, in the era of technical advances and modern software and hardware solutions, industries push surgeons toward using, promoting, and publicizing vitrectomy. On the other hand, the lower cost of buckling should be a motive to keep it alive and use it more often in properly selected cases with fresh RRD for the benefit of our patients [94,95].

## 4. Conclusions

Despite the constantly enlarging pool of available randomized clinical trials and meta-analysis data, the choice of the most preferable and successful primary surgical procedure for RRD repair still remains controversial and depends on surgeons’ preferences.

Studies reporting anatomic outcomes of PPV and SB surgery differ regarding lens status and type of retinal detachment recommendations. The number of PPV surgeries in the modern world significantly outgrows the number of SB procedures. Also, criteria for including RD patients in certain trials were inconsistent in terms of the “primary RD” definition in more than 65% of patients operated from retinal detachment. The rate of primary retinal reattachment (of approx. 70%) in both SB and PPV, as well as the rate of secondary reattachment (of approx. 95%), and stable 6-months postoperative visual acuity (VA) in pseudophakic and aphakic patients have been founded the same. Only mildly less (but statistically significant) 6-month VA in a group of phakic patients treated with PPV was noted due to cataract progression [96].

Surgical strategies for primary RRD repair in the 21st century should be based, in the first place, on the clinical aspect of retinal detachment itself. They should also include analysis of other factors, like the number, size, and location of retinal breaks, lens status, patient’s expected ability to adhere to required postoperative positioning, available operating room facilities, surgical equipment, and supporting background (anesthesiology service and educated nurses), previous experience and surgical preferences of the surgeon, and the last but not the least, personal aspect and lifestyle of the patient. The main goal, anatomic and functional recovery of the eye and retinal status, is achievable, as considered, for the greatest number of patients with primary RRD. 

In the eyes with RRD, where surgical procedure comprised of PPV with gas or silicon oil tamponade, but without lens surgery, acceleration of cataract is inevitable, in that way, making cataract progression one of the main disadvantages of a primary pars plana vitrectomy approach to RRD surgery. PPV with its peripheral endolaser retinal photocoagulation (either focal (peri rupture) or 360 degrees) is connected with a higher incidence of PVR progression and epiretinal membrane formation, especially in patients with advanced forms of RRD, in myopic eyes and in cases with systemic diseases like diabetes mellitus [97], autoimmune diseases, and immunodeficiency.

Scleral buckling procedure, either as encircling or sectorial episcleral implant, is often escorted with postoperative diplopia, ptosis, the refractive shift towards myopia (due to axial length enlargement), astigmatism (with segmental buckle), motility disorders, anterior-segment ischemia, local eye irritations, and sometimes even scleral implant or encircling band exposure with conjunctival scarring. Specific time after SB RRD surgery, scleral tissue can become very thin and eroded with an implant in cases of stronger indentation, leaving atrophic patches of sclera with uveal tissue visualization, almost creating staphyloma beneath it. Those parts of the eye are weak points in scleral integrity, therefore making possible rupturing sites in cases of more severe eye trauma or additional eye surgery [98]. 

Increased postoperative pain in RRD patients treated with scleral buckling procedure is documented due to more complicated and more intense surgical ocular tissue (especially ocular muscles) manipulation during scleral buckling surgery, making intra and postoperative pain one of the most significant disadvantages of SB surgery for RRD.

Still, SB surgery is nowadays again making a comeback to the retinal surgical armamentarium because years of predominance of PPV have shown that some cases of RRD cannot be solved only with intraocular procedures. Both PPV and SB surgery require surgical education and take time to perform in an effective enough way to achieve retinal detachment with a minor rate of complications and a number of reoperations needed. In practice, both scleral buckling and vitrectomy techniques require plenty of surgical skill and practice. Lower incidence of scleral buckling techniques in primary RRD repair can be connected to insufficient training of retinal surgeons for buckling procedures in modern times and, last but not least, to significantly lower reimbursement rates to eye units where retinal surgery is performed.

Like most surgical procedures in modern ophthalmology, pars plana vitrectomy surgery with vitreous removal has evolved over time. It now routinely includes subretinal fluid drainage, endo diathermy, air–fluid exchange, and gas or silicon oil tamponade. With the use of advanced instruments of smaller gauge, the average rate of retinal reattachment after the first RRD surgery is now about 85 to 90% of cases [67,99]. 

Modern instruments for pars plana surgery range from 23 to 25, 27, and even 29 gauges [100,101], tending to minimalize the size of the port, i.e., the wound created in the pars plana part of the sclera. Cutting the rate of updated combined platforms for phacoemulsification and vitrectomy surgery counts from 5000 to 20,000 cuts per minute (CPM) [102,103], allowing shorter surgical time and, most importantly, better fluidics with reduced traction to vitreous and retina. With improved duty cycle control, better efficiency and higher safety during retinal surgery are achieved [104].

Trained vitreoretinal surgeons may change the number of cuts, and level of suction (aspiration), and adjust the height of the bottle (and intraocular pressure), therefore influencing the cutting rate and traction to the peripheral retina, while lowering the risk of iatrogenic breaks. With the latest instruments of small gauge available and improved vitrectomy guillotine design, surgeons can come very close to a detached retina in order to remove most of the vitreous present, even in close pre-retinal space, without causing moving or damaging retina.

Changing surgical technique from 20 gauge and no valves and trocars to 23 to 25 gauge or smaller trocars with valves had a big influence by enabling better intraoperative intraocular pressure control [105,106]. During the frequent routine intraoperative exchange of instruments, valved trocars prevent fluid leakage and maintain globe pressure during vitrectomy. By using trocars and valves, the posterior eye is filled with infusion, and incidents like retinal incarceration, the collapse of the eyeball, the incidence of ocular hypotonia and resulting optic nerve ischemia, sudden suprachoroidal edema or expulsive suprachoroidal hemorrhage are by frequency brought to a minimum. In the case of aphakic eyes, valved trocars also influence stabilizing the anterior chamber with pars plana infusion. 

New 3D and heads-up visualizing systems (HUS) during retinal surgery enable easier work for the surgeon and follow-up of the surgical procedure by residents and the whole surgical team because the course of the surgery can be followed on big screens in operating theatres [107,108,109]. Advanced widefield systems are also available, and wide-field view of the retina and retinal periphery can be assessed even up to 100 degrees, even in poorer conditions like a small pupil or media opacities present.

## 5. Future Directions

When deciding on the proper method for primary rhegmatogenous retinal detachment (RRD) repair, we have a variety of techniques to choose from. Choosing a vitreoretinal surgeon is often not easy. Apart from a surgeon’s personal preference, there are always multifactor conditions to take into consideration. In the first place, the type of retinal break and detachment, the duration of the retinal detachment, and the age of the patient are important. 

Most retina specialists still debate about the advantages and disadvantages of specific RRD procedures. In most of the literature, with data on RRD surgery available, preoperative and postoperative status of anatomical and visual repairment were analyzed. Nevertheless which surgical method was used in particular, it appeared outcomes were case dependent. Decisions on the most appropriate surgical option for RRD patients should be adjusted to the patient’s retinal and general condition and age, not to the surgeon’s skills or preferences.

Bearing in mind that pars plana vitrectomy has (and will have) a dominant role in retinal detachment surgery in the modern world, we may, in addition, claim that scleral buckling is still not on the “endangered species list,” as has been reported several years ago and that it still has something to offer. The trends in the policy of ophthalmology wards and education of young retina specialists and future vitreoretinal surgeons must include training in the field of small gauge pars plana vitrectomy, but also buckling surgery and indirect ophthalmoscopy.

## Figures and Tables

**Table 1:** Features of two most common surgical methods for primary rhegmatogenous retinal detachment (RRD) repair.

Surgical Method for Primary RRD Surgery	Buckling Surgery	PPV
**Preferred Lens Status**	Phakic	Pseudophakic/Aphakic
**Location of retinal break**	No difference In inferior break, no gas tamponade is possible	Preferrably upper quadrants of retina Inferior breaks are difficult to manage with standard gas or standard silicon oil tamponade (additional buckle is preferrable or use of heavy density silicon oil)
**Release of VR traction**	Indirect	Direct
**Drainage of SRF**	From outside	From inside
**Postoperative positioning**	Required with intraocular gas tamponade	Usually required
**Postoperative inflammation**	Significant	Mild
**Postoperative pain**	Usually pronounced	Mild of moderate
**Surgical trauma**	Higher	Lower
**Advantages**	Preoperatively overlooked retinal tears covered by buckle or encircling band Early flight possible (depending on intraocular gas tamponade)	Use of valves/trocars Better visibility Faster retinal reattachment
**Complications**	Refractive changes (myopia (esp. encircling band) and astigmatismus (esp. segmental buckles) Motility restrictions and diplopia Cystoid macular edema or epiretinal membrane formation Scleral perforation Exudation after cryotherapy Vitreal or subretinal hemorrhage Dislocation or protrusion of buckle or encircling band Infection of the buckle Endophthalmitis	New iatrogenic retinal tears Intra- or postoperative rise in intraocular pressure Ocular hypotonia Cataract formation Cystoid macular edema or epiretinal membrane formation Difficulties with air/fluid exchange Difficulties with intraocular tamponade Remnants of intraocular tamponade (PFC or SO particles) Endophthalmitis
**Previous corneal surgery**	Possibility of impaired visualisation	Possibility of impaired visualization Flap slippage in flap-based corneal surgery
**Postoperative recovery time**	Multifactorial, Longer	Mutlifactorial, Shorter
**Dependance on devices**	Low	High
**Costs**	Low	High

## Data Availability

No new data were created or analyzed in this study.

## Abbreviations

RD	retinal detachment
RRD	rhegmatogenous retinal detachment
PR	pneumatic retinopexy
SB	scleral buckling
PPV	pars plana vitrectomy
SRF	subretinal fluid
VR	vitreoretinal
PVR	proliferative vitreoretinopathy
MIVS	minimal invasive vitrectomy surgery
OCT	intraoperative optic coherence tomography
SOSR	single-operation success rate
SSAS	single surgery anatomic success
RCTs	randomized clinical trials
HUS	heads up visualizing systems
